# The Relationship between Patellofemoral Pain Syndrome and Hip Biomechanics: A Systematic Review with Meta-Analysis

**DOI:** 10.3390/healthcare11010099

**Published:** 2022-12-28

**Authors:** Pingping Xie, Bíró István, Minjun Liang

**Affiliations:** 1 College of Science and Technology, Ningbo University, Ningbo 315211, China; 2 Faculty of Engineering, University of Szeged, 6720 Szeged, Hungary; 3 Faculty of Sports Science, Ningbo University, Ningbo 315211, China

**Keywords:** patellofemoral pain, proximal muscle strength, lower limb biomechanics, hip strength, gluteus muscle activation

## Abstract

(1) Background and purpose: Muscular control and motor function in a patient with Patellofemoral pain syndrome (PFPS) have not yet been investigated systematically. Therefore, this review synthesis the previous results about the association of PFPS with gluteus muscle activation, hip strength, and kinematic characteristic of the hip and knee joint, to deepen understanding of the PFPS etiology and promote the establishment of an effective treatment strategy. (2) Methods: A literature search was conducted from January 2000 to July 2022 in four electronic databases: Medline, Embase, Google scholar, and Scopus. A total of 846 articles were initially identified, and after the screening process based on the inclusion criteria, 12 articles were eventually included. Means and SDs of gluteus medius (GMed), gluteus maximus (GMax), hip strength, and kinematic variation of hip and knee were retrieved from the present study. (3) Results and conclusion: Regarding kinematic variation, moderate evidence indicates that an increased peak hip adduction was found in PFPS groups during running and single leg (SL) squat activities. There is no difference in the GMed and GMax activation levels between the two groups among the vast majority of functional activities. Most importantly, strong evidence suggests that hip strength is weaker in individuals with PFPS, showing less strength of hip external rotation and hip abduction compared to the control group. However, without prospective studies, it is difficult to determine whether hip strength weakness is a cause or a result of PFPS. Therefore, further research is needed to evaluate the hip strength level in identifying individuals most likely to associated with PFPS development is needed.

## 1. Introduction

PFPS has been described as one of the most perplexing and clinically challenging chronic disorders [1]. Symptoms usually include diffuse pain originating from the anterior aspect of the patella, and commonly along the medial aspect of the knee [2]. It therefore limits suffers’ daily activities that need loading on a flexed knee. There is a high incidence, especially among runners, with PFPS accounting for 46% of running-related injuries [3]. However, the etiology of this disorder remains vague and controversial [4,5]. This is reflected in the lack of consensus on how PFPS should be treated clinically.

Considerable research has focused on determining the root cause of PFPS, multiple factors have been thought to be the possible reason, with various intrinsic and extrinsic biomechanical characteristics involved. Particularly, one of the intrinsic factors that have received increasing attention in previous literature is that the abnormal femur kinematic is being contributed to altered patellofemoral joint (PFJ) mechanics [6]. This concept has been supported by the work of lee et al. who found that a 30° femur internal rotation significantly increased the PFJ contact pressure [7]. It also has been proposed that hip adduction could lead to knee valgus along with increased contact forces acting on the lateral facet of the patella [8]. Therefore, it is deemed that structural abnormalities in the hip movement have been implicated as a contributor to PFPS. 

On the other hand, the movement pattern is dominated by neuromuscular control via coordinating muscle activity, therefore, the relationship between the muscle function of the lower limbs and the PFPS deserves to be investigated comprehensively. It has been documented that proximal muscle weakness of the hip is associated with subsequent distal disorder [9]. Cichanowski et al. indicated that female patients with PFPS had significantly weaker hip abductors and external rotators than a health group [10]. In addition, the patella tracking disorder is also thought to be a possible reason for PFPS development, it is a result of the quadriceps muscle weakness and delayed activation of vastus medialis (VM) relative to the vastus lateralis (VL). Neptune et al. investigated that a 5 ms VM timing delay is related to a significantly increased loading in the lateral facet of the PFJ [11].

However, most studies have overlooked a combination role of muscular control and motor function in subjects with PFPS, this information would further enhance understanding of the etiology of PFPS development and establish an effective treatment strategy. Therefore, this review is systematically investigating the association of PFPS with gluteus muscle activation, hip strength, and kinematic characteristic of the hip and knee joints. The normal and abnormal muscular and kinematic functions of the lower limbs related to PFPS pathology will be reviewed within the previous literature.

## 2. Materials and Methods

### 2.1. Search Strategy and Inclusion Criteria

All selected papers were screened by two authors, and papers deemed appropriate were divided into one of four criteria:

1. Knee joint musculature strength in PFPS
  - Studies including subjects with a PFPS diagnosed
  - Studies evaluated the effects of knee muscular strengthening in subjects with PFPS
2. Hip joint musculature strength in PFPS
  - Studies including subjects with a PFPS diagnosed
  - Studies evaluated the effects of hip muscular strengthening in subjects with PFPS
3. Hip joint kinematic variation in PFPS
  - Studies including subjects with PFPS diagnosed
  - Studies examined the effects of hip joint kinematics on subjects with PFPS.
4. Knee joint kinematic variation in PFPS
  - Studies including subjects with PFPS diagnosed
  - Studies examined the effects of knee joint kinematic characteristics on subjects with PFPS.

### 2.2. Quality Assessment of Selected Studies

A modified version of The Downs and Black Quality Index (DBQI) was used to conduct the quality assessment of the included studies, it comprises 15 scale items, with good inter-rater reliability (r = 0.75) [12]. The quality of included studies has been divided into three classifications based on the score assessment, which are high quality (score ≥ 11), moderate quality (6 ≤ score ≤ 10), and low quality (score ≤ 5). Two independent authors examine the included studies, if there is any controversial opinion on the included study between the two authors, a discussion would be proceeded to reach a consensus. Inter-rater reliability of each included study was identified using percentage agreement.

### 2.3. Data Extraction and Analysis 

The sample sizes, muscle, kinetic, and kinematic variables of the hip and knee joint, function activity, and participant demographics (age, mass, height, and gender) were retrieved. The means and SDs of each parameter in the included study were extracted. Extracted data were pooled where at least two included studies evaluated the same variable. The statistical heterogeneity level of the pooled data was evaluated using χ^2^ and I^2^ statistics, the heterogeneity was defined as *p* < 0.05. "Level of evidence" is defined as suggested by Van Tulder et al. including four categories: “strong evidence”, “moderate evidence”, limited evidence”, and “very limited evidence” [13].

## 3. Results

### 3.1. Search Results 

**Figure 1:**
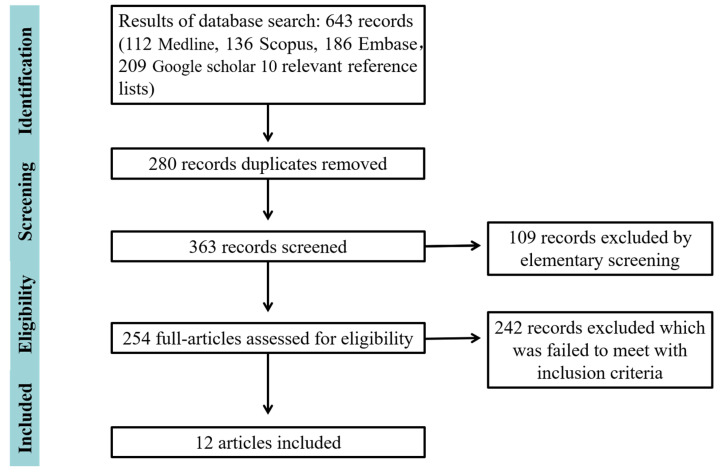
The screening process of included studies.

A total of 643 articles were initially determined via four electronic databases, and 10 extra articles were identified from the relative reference list. According to the inclusion criteria, 280 duplicate studies and 15 conference articles were excluded; 241 articles were retained for the full-text assessment after a screening of titles and abstracts. Finally, 12 articles were included for systematic review, of those, no prospective studies were identified. The whole process of searching and screening is presented in Figure 1.

### 3.2. Quality Assessment of Included Studies

As shown in Table 1, the score range of the included studies is based on the Downs and Black scale, in which 9 articles were identified as high quality with scoring between 11–14, and 4 articles were rated as moderate quality with a scoring between 6 and 10.

### 3.3. Studies Characteristic

Study characteristics are shown in Table 1, including sample sizes of gender and participant demographics from included studies. In addition, function activity, pain duration, muscle activation, kinetic, and kinematic variables can be found in Table 2 and Table 3.

### 3.4. Kinematic Variation

**Figure 2:**
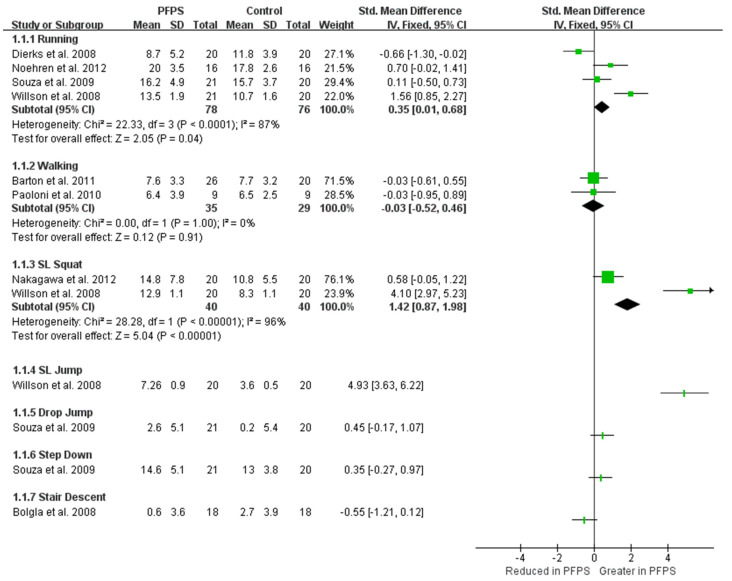
Hip adduction among the variety of function activities [14,15,16,17,18,19,20,21].

**Figure 3:**
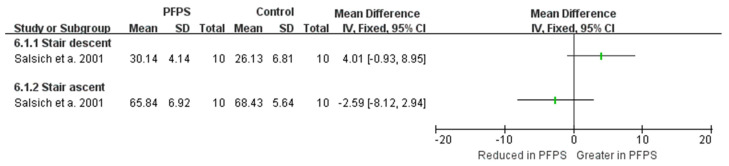
Hip flexion angle between the PFPS and control in different functional activities [22].

In terms of kinematic findings of hip adduction and hip flexion, among the various function activities, can be found in Figure 2 and Figure 3. There is moderate evidence from two high-quality studies and two moderate-quality studies exhibited that the PFPS subject has a greater hip adduction angle than the controlled subject in running activity [I^2^ = 87%, small significant, SMD = 0.35 (0.01–0.68)] [14,15,16,17]. Also, moderate evidence from one high-quality study and one moderate-quality study indicates a greater hip adduction angle in PFPS than in controlled subject during single leg (SL) squat [I^2^ = 96%, large significant, SMD = 1.42 (0.87–1.98)] [17,18]. Moderate evidence from one high-quality study [19], and from one moderate-quality study shows no difference in hip adduction during walking between the two groups [20]. Additionally, a single moderate-quality study indicates limited evidence that PHPS has higher hip adduction than controlled SL jump activity [17], but no difference was found in drop jump and step-down activities [15]. One single high-quality study indicates limited evidence that hip adduction was reduced in PFPS than in controlled subjects [21]. Single limited evidence indicates that hip flexion value in PFPS subjects was increased in stair descent, but reduced in stair ascent activity [22].

**Figure 4:**
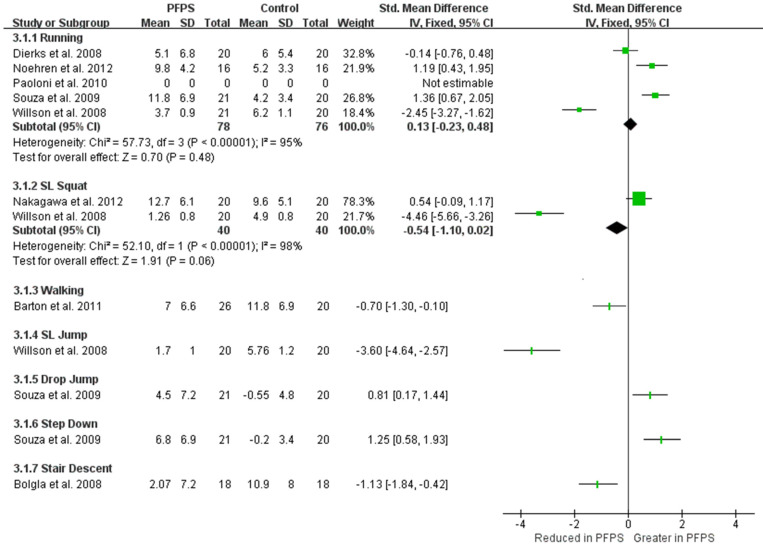
Hip internal rotation angle between PFPS and control group among the various functional activities [14,15,16,17,18,19,20,21].

Hip internal rotation between PFPS and controlled subject among various functional activities can be found in Figure 4. Moderate evidence from two high-quality studies [14,16] and three moderate-quality studies showed no difference in hip internal rotation during running between PFPS and control groups (I^2^ = 95) [15,17,20]. Limited evidence from one high-quality study and from one moderate-quality study indicate that there was no difference in hip internal rotation between the two groups (I^2^ = 98%) in SL squats [17,18]. In addition, there is limited evidence showing an inconsistent result of hip internal rotation value among the five different functional activities between PFPS and control groups, such as a reduced hip internal rotation in PFPS was identified in walking and stair descent activities by two single high-quality studies [19,21], and an increased hip internal rotation in PFPS was found in drop jump and step-down activities by one single moderate quality study [15].

**Figure 5:**
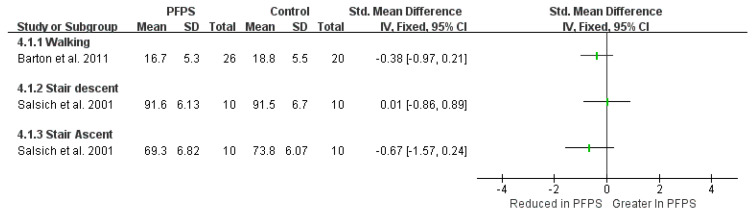
Knee flexion angle among the various functional activities [19,22].

**Figure 6:**
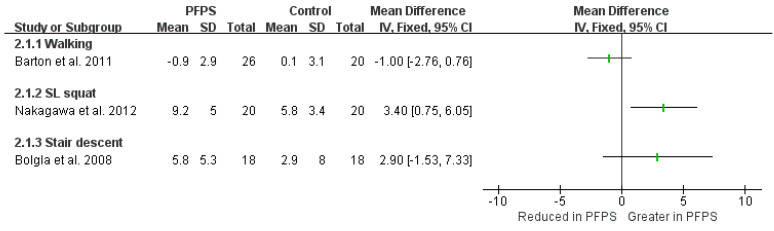
Knee abduction angle between the PFPS and control among the various functional activities [18,19,21].

**Figure 7:**
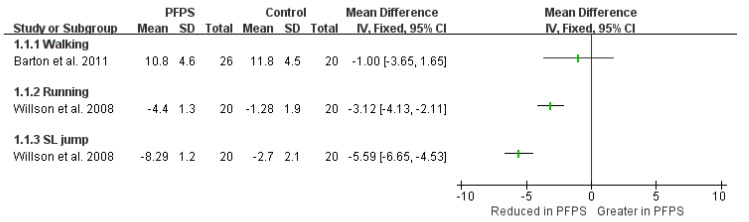
Knee internal rotation angle between the PFPS and control group among the various functional activities. 3.5. Hip strength and torque [17,19].

There is no pooled result regarding knee kinematics between PFPS and control groups due to the insufficient data, and only limited evidence can be obtained on the basis of a single study (Figure 5, Figure 6 and Figure 7). In terms of knee flexion, a reduction value has been found in PFPS compared to the control group in one high-quality study conducted walking activity [19], and one moderate-quality study conducted in a stair ascent activity [22], no difference was found in the stair descent activity [22]. For knee abduction, one single high-quality study of walking activity indicates a smaller value in PFPS [19], and two single high-quality studies of SL squat and stair descent activities indicate a greater value in PFPS [18,21]. For knee internal rotation, one single high-quality and one moderate-quality study indicates a reduction value in PFPS when compared to the control group in walking, running, and SL jump activity [17,19].

### 3.5. Hip Strength and Torque

**Figure 8:**
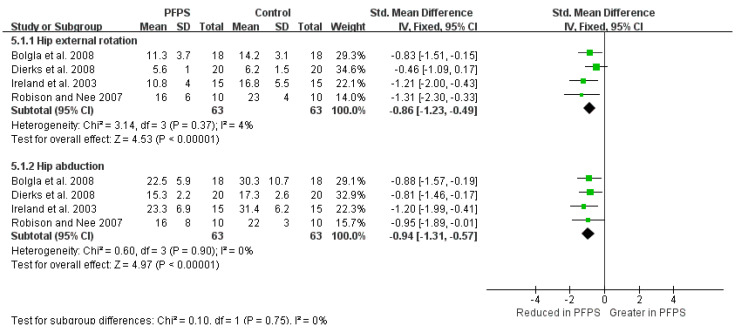
Hip strength in isometric strength testing [14,21,23,24].

**Figure 9:**
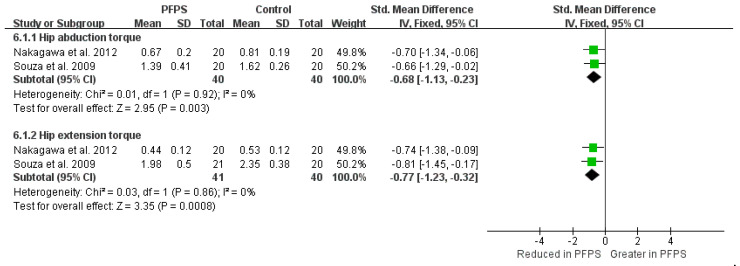
Hip torque [15,18].

The Strong evidence from four high-quality studies indicates a reduction value of hip external rotation strength in PFPS [I^2^ = 4%, small significant, SMD= −0.86 (−1.23 to −0.49)], also a reduction value of hip abduction strength in PFPS [I^2^ = 34%, small significant, SMD = −0.94 (−1.31 to −0.57)] [14,21,23,24] (Figure 8). Furthermore, in terms of hip torque, moderate evidence from one high-quality study and one moderate-quality study indicates a reduced hip abduction torque [I^2^ = 35%, small significant, SMD = −0.68 (−1.13 to −0.23)] [14,21,23,24], and a reduced hip extension torque [I^2^ = 35%, small significant, SMD = −0.77 (−1.23 to −0.32)] in PFPS compared to the controlled subject (Figure 9).

### 3.6. Muscle Activation

**Figure 10:**
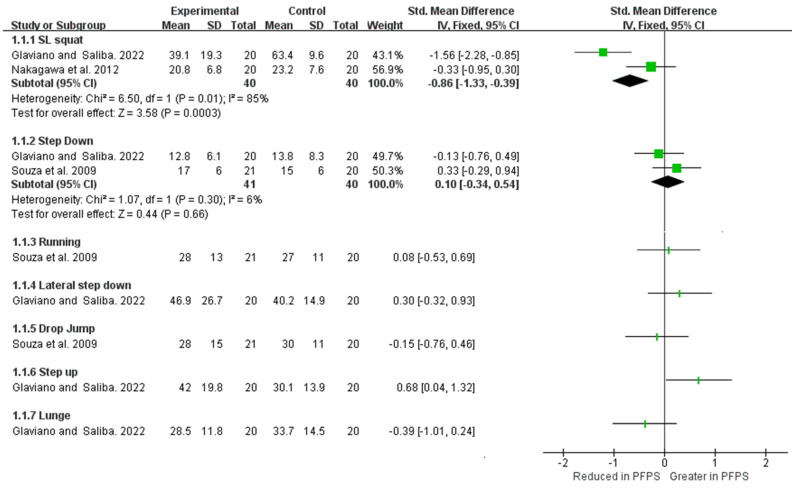
GMed activation among the functional activities [15,18,25].

**Figure 11:**
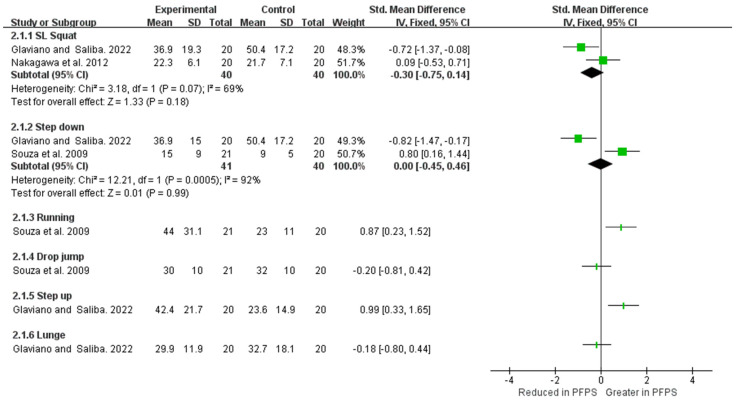
GMax activation between PFPS and control group among the various functional activities [15,18,25].

As shown in Figure 10 and Figure 11. In the SL squat activity, strong evidence from two high-quality studies indicates a reduction value of GMed activation in PFPS compared the to control group [I^2^ = 85%, small significant, SMD = −0.86 (−1.33 to −0.39)], but no difference was found in GMax activation [I^2^ = 69%, SMD = −0.3 (−0.75 to −0.14)] [18,25]. In the step-down activity, moderate evidence from one high-quality study and one moderate-quality study shows no difference in GMed activation [I^2^ = 6%, SMD = 0.1 (−0.34 to 0.54)] and GMax activation [I^2^ = 92%, SMD = 0 (−0.45 to 0.46)] between two groups. Limited evidence from a single study demonstrates no difference in GMed activation between the two groups in running, lateral step down, and drop jump activity [15,25]. Limited evidence from one single high-quality study indicates a higher and lower GMed activation in step-up and lunge tasks respectively [25]. Limited evidence from one single moderate-quality study and one single high-quality study indicates no difference in GMax activation in drop jump and lunge activity between the two groups, but higher GMax activation in PFPS during running and step-up activities [15,25].

## 4. Discussion

This systematic review identified 12 published articles evaluating muscle activation, hip strength, and kinematic characteristics associated with PFPS among the various functional activities. There is currently demonstrated moderate to strong evidence that no significant difference in hip and knee kinematics was found between PFPS and control groups, except for moderate evidence identified the small and moderate significant association of PFSP with increased peak hip adduction in running and SL squat activities. PFPS was also associated with reduced strength and torque of hip internal rotation and hip abduction compared with controls. In addition, moderate evidence indicates that only a small significant association between PFPS and reduced GMed activation was identified during SL squat activity, but there is an association between PFPS and GMax activation in all measured activities.

Nine included studies that evaluated the peak value for hip kinematics during various functional tasks and seven evaluated knee kinematics. Only peak hip adduction was found to significantly differ, with moderate evidence indicating an increased peak value during running and SL squat tasks. This result in the running task is consistent with a systematic review that investigated the kinematic gait characteristics related to the PFPS [25]. Conceptually, excessive hip adduction angle in the PFPS subject could increase the dynamic Q angle which may lead to higher patellofemoral joint stress [26]. It has been identified by cadaveric simulations that increased Q angle contributed to greater compression force on the lateral aspect of the patella. Particularly, excessive hip adduction in female runners could be considered an important contribution to increase knee joint stress in running where there is repetitive exposure to high loads [25]. But this kinematic pattern is not consistent across all studies and functional activities, such as moderate evidence derived from the two included studies shows no difference in the walking task, also single studies showed no difference in the SL jump and step-down task. The discrepancy in results among various functional tasks may be attributable to a different method, such as the use of different kinematic models, as we observed that the vast majority of kinematic measurements were different in the included studies. About hip rotation during running and SL squat, there was no difference between the PFPS and controlled subject according to the pooled results. This finding is inconsistent with Arazpour et al. as they concluded a controversial consequence of hip rotation in PFPS subjects based on several studies, suggesting it is relevant to differences in the methodology factor, data analysis method, and participant selection [27]. Fundamentally, they were unable to perform the mate analysis to integrate data and provide a synthesis result. Also, we must admit that we failed to show the pooled results of knee kinematics among the various functional tasks because few studies were identified based on the inclusion criteria.

There is strong evidence that demonstrated a significant association between PFPS and hip muscle weakness with less strength of hip external rotation and hip abduction. Among the included studies, Ireland et al. first identified hip external rotator and abductor weakness in female patients with PSPF. Especially, they identified a reduction of 36% in hip external rotator strength and 26% in hip abductor strength in subjects with PFPS than those controlled [23]. The repetitive femur excessive adduction and internal rotation during movement could lead to lateral patella tracking and increased contact pressure in the later aspect of the patella. Consequently, the patella alignment could increase the incidence of PFPS development [28,29,30]. In general, the results of weak hip strength in PFPS subjects were consistent among the included studies, which all used similar methods to assess hip muscle strength, such as test equipment, standard test positions, and handheld dynamometer placement. The standardized methodology has enabled the ability to make a meaningful conclusion. However, caution must be taken when considering improving hip muscle strength as a clinical treatment strategy for PFPS patients, because without prospective studies investigating the PFPS development in the patient, it is difficult to define whether the hip weakness is a precursor of PFPS or whether it is a result of disuse atrophy or motor control after PFPS [23]. On the other hand, previous studies demonstrated the interrelationship between hip weakness and altered lower limb kinematics [31,32]. However, according to the currently pooled results of hip and knee kinematic characteristics, only a small significant association of PFPS and hip adduction was found during running and SL squat activities, suggesting that hip muscle weakness may not necessarily lead to a change in hip and knee kinematic. In addition, it may have to do with the fact that the chosen task is not challenging enough, such as stair descent, step down, walking, lunge, and drop jump.

What’s more, one of the important factors related to hip strength in PFPS patients should be mentioned, such as femoral anteversion, which is thought to have a direct impact on patellar tracking, and excessive femoral anteversion is significantly associated with the increased patellofemoral contact pressure. Nyland et al. demonstrated that subjects with increased femoral anteversion caused a reduction in the peak hip abductor with peak glutes medius EMG amplitude decreased by 34% and peak vastus medialis EMG decreased by 27% in isometric functional measurement [33]. Femoral abnormal alignment with the disturbed muscular function of the hip joint directly influences the joint stability of the lower extremity. Besides, it has been suggested that femoral anteversion could cause an increase in the Q angle. Nonetheless, a greater Q angle would lead to a larger lateral vector and increase lateral patellar tracking compared to the smaller Q angle [32]. Unfortunately, the parameter of the femoral anteversion was not included here, due to this systematic review was mainly focused on the dynamic activities under weight-bearing. Also, in the published systematic reviews which included the factors related to PFPS, such as Lankhorst et al. examined 523 variables including 47 studies, but femoral anteversion was still ignored [34]. Considering the presence of increased femoral anteversion was very common in females, which would pose a higher potential risk for PFPS development, therefore, this factor deserves higher attention. 

There is no difference in GMed and GMax muscle activation between the two groups for the various functional activities, except a small significant association of PFPS and a reduction in GMed activation in SL squat. This result is coordinated with a previously published systematic review, in which the relationship between gluteal muscle activity and PFPS has been investigated, they suggested that the gluteal muscle activation level is less important in the pathology of PFPS. Moreover, they found that delayed and shorter duration of GMed respect with GMax is significantly related to PFPS [10]. Similarly, a previous study indicated the same GMed activation level between the health and PFPS group in the functional activity of running, drop jumping, and stepping down, concluding that there is compensated strategy in the PFPS patients whose trunk lean over the ipsilateral hip could minimize the gluteus muscle force to stabilize the pelvis [35]. In this research, we only focused on the gluteus muscle activation during various functional activities. The association of PFPS with quadriceps activation has received wide attention, and clinicians historically prescribed quadriceps exercise in PFPS patients. Systematically, Fagan and Delahunt have summarized that quadriceps retraining provided good clinical outcomes in patients with PFPS [9]. Additional information in terms of the interrelationship between hip muscle function and knee joint biomechanics deserves further investigation.

Overall, the association between hip strength deficits and PFPS can be demonstrated based on the pooled findings, even though it is still vague to identify whether it is an initial cause or a final result of PFPS, hip strengthening as an incorporation strategy in the PFPS treatment is recommended. As previous randomized controlled trials have indicated that hip strengthening could further benefit in relieving patellofemoral pain and optimizing function in female patients with PFPS [36,37,38,39,40].

Some limitations existed in the current research, such as the kinematic measurement being widely different among the included studies, which may cause result bias when comparing kinematic data with each other in a specific functional activity. Also, we did not critique kinematic models used in the included studies, because there is currently a lack of effective evaluation tools to do so. Indeed, this is a paramount consideration in future kinematic measurements. The majority of participants are female in the included studies, in which more than half studies only investigated the difference between the two groups of female subjects. Although there is a higher incidence of PFPS development in females than males, the biomechanical mechanisms of PFPS should be included equally in female and male subjects in general use. Therefore, the sex bias must be considered when interpreting pooled results of this review.

## 5. Conclusions

The current systematic review investigated the association of PFPS and hip strength, gluteus muscle activation, and kinematic variation of hip and knee joints. There is no difference in the GMed and GMax activation levels between the two groups among the vast majority of functional activities. Regarding kinematic variation, moderate evidence indicates that an increased peak hip adduction was found in PFPS groups during running and SL squat activity. Most importantly, strong evidence suggests that hip strength is weaker in individuals with PFPS, showing less strength of hip external rotation and hip abduction, also less hip external rotator, and less hip abductor compared to the control group. However, without prospective studies, it is difficult to determine whether hip strength weakness is a cause or a result of PFPS. Therefore, further research is needed to evaluate the hip strength level in identifying individuals most likely to associated with PFPS development is needed.

## Figures and Tables

**Table 1:** Quality assessment of included studies.

Study	1	2	3	5	6	7	10	11	12	15	16	18	20	21	25	Total
Dierks et al. 2008 [14]	1	1	1	1	1	1	1	0	U	U	1	1	1	1	U	11
Lack et al. 2009 [15]	1	1	1	0	1	1	1	0	U	U	1	1	1	1	U	10
Noehren et al. 2012 [16]	1	1	1	1	1	1	1	0	U	U	1	1	1	1	U	11
Willson et al. 2008 [17]	1	1	0	1	1	1	1	0	U	U	1	1	U	1	U	9
Nakagawa et al. 2012 [18]	1	1	1	1	1	1	1	0	U	U	1	1	1	1	U	11
Barton et al. 2011 [19]	1	1	1	1	1	1	1	0	U	U	1	1	1	1	U	11
Paoloni et al. 2010 [20]	1	1	0	1	1	1	1	0	U	U	1	1	0	1	U	9
Bolgla et al. 2008 [21]	1	1	1	1	1	1	1	0	U	U	1	1	1	1	U	11
Salsich et a. 2001 [22]	1	1	1	1	1	1	1	0	U	U	1	1	U	1	U	10
Ireland et al. 2003 [23]	1	1	1	1	1	1	1	0	1	U	1	1	0	1	U	11
Robinson and Nee 2007 [24]	1	1	1	1	1	1	1	0	U	U	1	1	1	1	U	11
Glaviano and Saliba 2022 [25]	1	1	1	1	1	1	1	0	U	U	1	1	1	1	U	11

Item 5 is defined as yes = 2, partial = 1, no = 0, U = unclear. All other items are assessed as yes = 1, no = 0, and U = unclear.

**Table 2:** Specific details about sample sizes, participant demographics, and population source of included studies.

Study	Sample Size		Gender (F:M)		Age Range (Mean Age)		Height (m), Weight (kg)	
	PFPS	CON	PFPS	CON	PFPS	CON	PFPS	CON
Dierks et al. 2008 [14]	20	20	15:5	15:5	NR (24.1 ± 7.4)	NR (22.7 ± 5.6)	1.71 ± 0.1 m 65.75 ± 12.56 kg	1.70 ± 0.08 m 63.02 ± 9.15 kg
Lack et al. 2009 [15]	21	20	21:0	20:0	18-45 (27 ± 6)	18-45 (26 ± 5)	1.70 ± 0.06 m 65 ± 10 kg	1.70 ± 0.05 m 63 ± 7.0 kg
Noehren et al. 2012 [16]	16	16	16:0	16:0	18–45 (27 ± 6)	18–45 (25 ± 4)	1.64 ± 0.05 m 57.4 ± 4.6 kg	1.65 ± 0.07 m 58.7 ± 6.5 kg
Willson et al. 2008 [17]	20	20	20:0	20:0	NR (23.3 ± 3.1)	NR (23.7 ± 3.6)	1.66 ± 0.08 m 61.7 ± 10.6 kg	1.66 ± 0.06 m 61.1 ± 5.4 kg
Nakagawa et al. 2012 [18]	20	20	20:20	20:20	F: NR (22.3 ± 3.1) M: NR (24.2 ± 4.4)	F: NR (21.8 ± 2.6) M: NR (23.5 ± 3.8)	F: 1.66 ± 0.59 m 61.1 ± 7.5 kg M: 1.80 ± 0.51 m 77.0 ± 9.6 kg	F: 1.63 ± 0.73 m 59.4 ± 7.3 kg M: 1.76 ± 0.6 m 74.6 ± 9.1kg
Barton et al. 2011 [19]	26	20	21:5	16:4	18–35 (25.1 ± 4.6)	18–35 (23.4 ± 2.3)	1.6 ± 8.4 m 66.7 ± 12.8 kg	1.7 ± 8.4 m 66.0 ± 15.4 kg
Paoloni et al. 2010 [20]	9	9	7:2	7:2	19–45 (28.1 ± 8.1)	21–38 (18.3 ± 5.9)	1.71 ± 0.09 m 64.4 ± 9.5 kg	1.70 ± 0.09 m 64.2 ± 10.8 kg
Bolgla et al. 2008 [21]	18	18	18:0	18:0	NR (24.5 ± 3.2)	NR (23.9 ± 2.8)	1.7 ± 0.1m 63.1 ± 9.1kg	1.7 ± 0.1m 62.1 ± 8.5 kg
Salsich et a. 2001 [22]	10	10	5:5	5:5	22–55 (36.5 ± 11.1)	21–42 (31.9 ± 7.3)	1.73 ± 10.3 m 70.9 ± 13.3 kg	1.70 ± 11.3 m 14.5 ± 67.7 kg
Ireland et al. 2003 [23]	15	15	15:0	15:0	12–21 (15.7 ± 2.7)	12–21 (15.7 ± 2.7)	63.1 ± 16.5 kg	56.6 ± 12.5 kg
Robinson and Nee 2007 [24]	10	10	10:0	10:0	12–34 (21.0)	16–35 (26.6)	63.5 kg	66.5 kg
Glaviano and Saliba 2022 [25]	20	20	20:0	20:0	NR (21.3 ± 2.7)	NR (20.7 ± 2.1)	1.68 ± 6.4 m 20.7 ± 21.0 kg	1.67 ± 6.5 m 64.2 ± 9.5 kg

NR = no report; F = female; M = male; CON = control.

**Table 3:** Participant activities, muscles, and kinematics variables were evaluated in each included study.

Study	Functional Activity	Pain Duration	Muscle Strength	Muscles	EMG Variable	Kinematics (Peak)
Dierks et al. 2008 [14]	Running; IST	More than 2 months	Hip abductor: Hip external rotator			
Lack et al. 2009 [15]	Running Stair descent DLDJ			GMed GMax	The average magnitude of activity (%MVC - average over stance period)	Peak hip rotation Peak hip adduction Peak hip torque
Noehren et al. 2012 [16]	Walking	More than 6 weeks				Knee flexion; abduction; internal rotation Hip adduction; internal rotation
Willson et al. 2008 [17]	Stair descent Stair ascent	NR				Knee flexion Hip flexion
Nakagawa et al. 2012 [18]	SL squat	14.4 ± 12.8 months		GMed VM VL	Maximal voluntary isometric contraction (MVIC)	Peak hip adduction Hip internal rotation Knee abduction
Barton et al. 2011 [19]	walking	More than 6 weeks				Hip, knee, rearfoot, and forefoot movement in three planes
Paoloni et al. 2010 [20]	Single leg squat Single leg jump Running	NR				Knee internal rotation Hip internal rotation Hip adduction
Bolgla et al. 2008 [21]	IST Stair descent	14.4 ± 12.8 months	Hip abductor: Hip external rotator	-	-	Hip internal rotation Hip adduction Knee varus
Salsich et a. 2001 [22]	IST running	More than 2 months	Hip abductor: Hip external rotator			Knee adduction Hip internal rotation Hip adduction
Ireland et al. 2003 [23]	IST	More than 3 three months	Hip abduction Hip external rotation			
Robinson and Nee 2007 [24]	Running	More than 2 months				Hip adduction Hip internal rotation
Glaviano and Saliba 2022 [25]	SL squat Step up; Step down Lateral step-down Lunge	More than 3 months		GMed GMax VMO VL		-

IST = Isometric strength testing; GMed = Gluteus medius; GMax = Gluteus maximus; VM = vastus medialis; VL = vastus lateralis; DLDJ = Double leg drops jump landing; SL = single leg.

## Data Availability

Not applicable.
